# Lack of seroresponse to SARS-CoV-2 booster vaccines given early post-transplant in patients primed pre-transplantation

**DOI:** 10.3389/fimmu.2022.1083167

**Published:** 2023-01-16

**Authors:** Sarah Gleeson, Paul Martin, Tina Thomson, Katrina J. Spensley, Dawn Goodall, Rachna Bedi, Amarpreet Kaur Thind, Charlotte Seneschall, Jaslyn Gan, Stephen McAdoo, Liz Lightstone, Peter Kelleher, Maria Prendecki, Michelle Willicombe

**Affiliations:** ^1^ Centre for Inflammatory Disease, Department of Immunology and Inflammation, Imperial College London, London, United Kingdom; ^2^ Imperial College Renal and Transplant Centre, Imperial College Healthcare National Healthcare Service Trust, Hammersmith Hospital, London, United Kingdom; ^3^ Department of Infection and Immunity Sciences Northwest London Pathology NHS Trust, Charing Cross Hospital, London, United Kingdom; ^4^ Department of Infectious Diseases, Imperial College London, London, United Kingdom

**Keywords:** SARS-CoV-2, COVID- 19, immunosuppressed, kidney transplant, vaccine, infection

## Abstract

SARS-CoV-2 vaccines are recommended pre-transplantation, however, waning immunity and evolving variants mandate booster doses. Currently there no data to inform the optimal timing of booster doses post-transplant, in patients primed pre-transplant. We investigated serial serological samples in 204 transplant recipients who received 2 or 3 SARS-CoV-2 vaccines pre-transplant. Spike protein antibody concentrations, [anti-S], were measured on the day of transplantation and following booster doses post-transplant. In infection-naïve patients, post-booster [anti-S] did not change when V3 (1^st^ booster) was given at 116(78-150) days post-transplant, falling from 122(32-574) to 111(34-682) BAU/ml, p=0.78. Similarly, in infection-experienced patients, [anti-S] on Day-0 and post-V3 were 1090(133-3667) and 2207(650-5618) BAU/ml respectively, p=0.26. In patients remaining infection-naïve, [anti-S] increased post-V4 (as 2^nd^ booster) when given at 226(208-295) days post-transplant, rising from 97(34-1074) to 5134(229-5680) BAU/ml, p=0.0016. Whilst in patients who had 3 vaccines pre-transplant, who received V4 (as 1^st^ booster) at 82(49-101) days post-transplant, [anti-S] did not change, falling from 981(396-2666) to 871(242-2092) BAU/ml, p=0.62. Overall, infection pre-transplant and [anti-S] at the time of transplantation predicted post-transplant infection risk. As [Anti-S] fail to respond to SARS-CoV-2 booster vaccines given early post-transplant, passive immunity may be beneficial to protect patients during this period.

## Introduction

The COVID-19 pandemic brought significant disruption to transplantation globally, and as services started to resume, the safety of recipients was and has remained a priority for the community (1–3). For the general population, vaccination heralded both protection against severe morbidity and mortality, and a change of attitude towards the pandemic. However, with evolving variants and relatively short-term protection afforded by each inoculation, more vulnerable people within the population remain reliant on ‘booster’ vaccines and effective treatments (4–6). The optimal timing of vaccination and boosters for people undergoing transplantation and commencing immunosuppression remains a challenge.

From the extensive vaccine immunogenicity and effectiveness data which have emerged, it is now acknowledged that established kidney transplant recipients mount suboptimal immune responses to SARS-CoV-2 vaccines, and remain at enhanced risk of severe infection compared with vaccinated counterparts in the general population (7, 8). Hypo-responsiveness to SARS-CoV-2 vaccines is greatest when vaccination occurs in the first year after transplantation; the period when immunosuppression burden is the greatest and infection risk from all pathogens is at its highest (3, 7). Hence, it is generally recommended that potential solid organ transplant recipients are vaccinated pre-immunosuppression (9). However, for old (eg. influenza) and new (eg. SARS-CoV-2) pathogens which require repeat inoculations due to waning immunoprotection or evolving variants, evidence for the optimal timing for vaccination post-transplant is weak (10).

This study aims to describe the longitudinal immunogenicity of the SARS-CoV-2 vaccines in new transplant recipients who received at least 2 vaccine doses pre-transplant. We aim to assess response to booster doses in the post-transplant period, and report effectiveness outcomes in terms of infection and severity.

## Methods

### Patient selection

**Figure 1:**
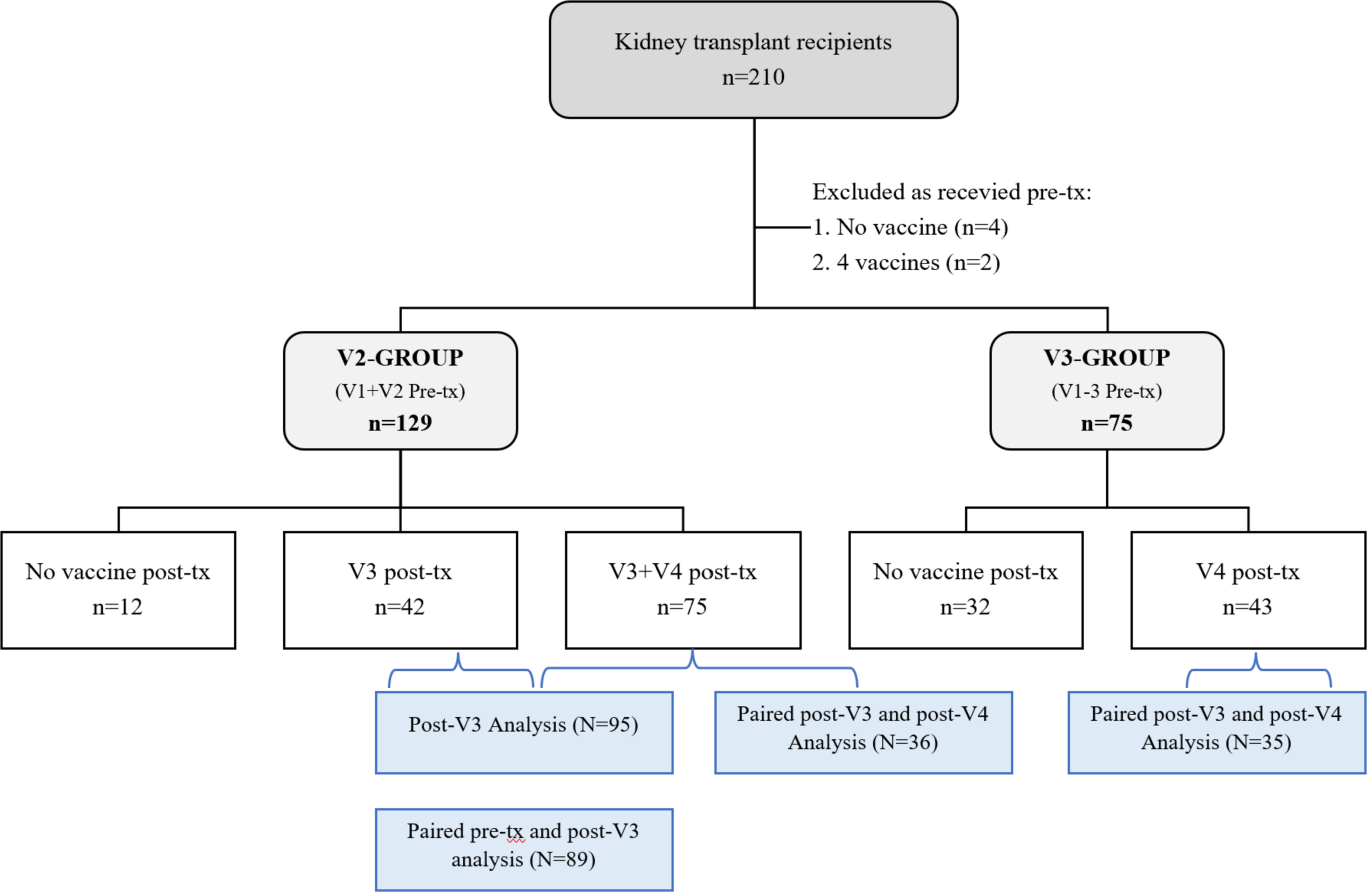
Patient cohort by vaccine status at the time of transplant^1^. ^1^Samples available for analysis as described.

All patients who received a kidney transplant between April 2021 and March 2022 at Imperial College Renal and Transplant Centre London were assessed for inclusion. Patients were eligible for participation if they had received 2 or 3 doses of a SARS-CoV-2 vaccine pre-transplantation, **Figure 1**. During the study period, it was routine clinical practice for patients to have SARS-CoV-2 serological assessment at the time of transplantation. Additional assessments were also undertaken following booster doses of vaccine. For the purposes of this report, only serological assessments performed between days 10-62 post-boost were considered. The median follow up of the study patients post-transplant was 11 (8–14) months. A cohort of haemodialysis patients receiving vaccination whilst awaiting a kidney transplant were used as controls, **Supplemental Information, Table S1**. Clinical and vaccine data were obtained from electronic patient records and vaccine database, respectively. The study was approved by the Health Research Authority, Research Ethics Committee (Reference: 20/WA/0123).

The immunosuppression protocol at our centre consists of monoclonal antibody induction for all patients with maintenance tacrolimus together with a steroid minimisation protocol. Patients receiving long-term corticosteroid treatment at the time of transplant are continued on their established dose post-transplant. In addition, maintenance mycophenolate mofetil (MMF) is prescribed for patients who received basiliximab or who are highly HLA sensitised, with a calculated reaction frequency of ≥85%.

### Definition of infection and detection of antibodies

Infection was confirmed through viral detection *via* reverse-transcriptase polymerase chain reaction (RT-PCR) assays, or by positive lateral flow antigen tests from April 2022. Infection outcome data was obtained from centrally collected data held by the National Health Service Blood and Transplant (NHSBT) Service *via* The UK Health Security Agency (UKHSA). Prior infection was defined by a history of viral detection or *via* serological assessment; nucleocapsid protein antibodies (anti-NP) at any time, or the presence of spike protein antibodies (anti-S) pre-vaccination. Anti-NP was tested using the Abbott Architect SARS-CoV-2 IgG 2 step chemiluminescent immunoassay (CMIA). Samples were interpreted as positive or negative with a threshold index value of 1.4. Anti-S IgG concentrations ([anti-S]) were assessed using the Abbott Architect SARS-CoV-2 IgG Quant II CMIA. A quantitative assay with a threshold value of 7.1 BAU/ml for positivity, and an upper level of detection of 5680 BAU/ml. Boosted status was defined as a post-vaccination level ≥700 BAU/ml, based on arbitrary internal observations of lower-level limits in healthy populations. Patients who were diagnosed with infection post-vaccination but prior to serological testing, were considered as infection-experienced.

### COVID-19 therapeutics

Treatment of COVID-19 infection was clinically based, and aligned with the UK Chief Medical Officer’s Interim Clinical Commissioning Policies (11). Prior to 20^th^ December 2021, all treatment was in-patient based, and the monoclonal antibody (mAb) utilised in algorithms was casirivimab plus imdevimab (Ronapreve^®^) in seronegative patients (2.4g). From 20^th^ December 2021, the mAb sotrovimab (Xevudy^®^) at 500mg doses, was available for people considered at highest risk of progression to severe disease in the community. Other outpatient treatments available during this period for this population included remdesivir, whilst for inpatients dexamethasone and tocilizumab were also considered.

### Statistical analysis

Statistical analysis was conducted using Prism 9.0 (GraphPad Software Inc., San Diego, California). Unless otherwise stated, all data are reported as median with interquartile range (IQR). The Chi-squared test was used for proportional assessments. The Mann-Whitney and Kruskal-Wallis tests were used to assess the difference between 2 or >2 groups, with Dunn’s *post-hoc* test to compare individual groups. Comparison of paired samples was assessed using the Wilcoxon test. Survival analysis was assessed using the log-rank test. A p value of <0.05 was deemed statistically significant.

## Results

Two-hundred and four patients were included; 129(63.2%) had received two vaccines (V2-group), and 75(36.7%) had received three vaccines (V3-group) pre-transplant, **Figure 1A** comparison of the V2-group and V3-group patient characteristics is shown in **Table 1**. Notably, the median time to transplantation following last vaccination was significantly longer in the V2-group compared with the V3-group; 103 (61–153) and 64(43-111) days respectively, p<0.001. At the time of transplantation, 90(69.8%) V2-group and 24(32.0%) V3-group patients were infection-naïve, p<0.0001.

**Table 1:** Characteristics of transplant patients.

Characteristics		All patients N=204 (%)	V2-Group N=129 (%)	V3-Group N=75 (%)	P value
**Gender**	Male	123 (60.3)	74 (57.4)	49 (65.3)	0.26
	Female	81 (39.7)	55 (42.6)	26 (34.7)	
**Age at transplantation**	Years (Median)	56 (45-65)	55 (45-65)	56 (45-66)	0.78
**Ethnicity**	White	69 (33.8)	38 (29.5)	31 (41.3)	0.24
	Black	25 (12.3)	18 (14.0)	7 (9.3)	
	Indoasian	83 (40.7)	53 (41.1)	30 (40.0)	
	Other	27 (13.2)	20 (15.5)	7 (9.3)	
**Cause of ESKD**	Polycystic kidney disease	16 (7.8)	13 (10.1)	3 (4.0)	0.07
	Glomerulonephritis	51 (25.0)	26 (20.2)	25 (33.3)	
	Diabetic nephropathy	62 (30.4)	45 (34.9)	17 (22.7)	
	Urological	17 (8.3)	8 (6.2)	9 (12.0)	
	Unknown	41 (20.1)	27 (20.9)	14 (18.7)	
	Other	17 (8.3)	10 (7.8)	7 (9.3)	
**Number of transplants received**	1	170 (83.3)	106 (82.2)	64 (85.3)	0.56
	≥2	34 (16.7)	23 (17.8)	11 (14.7)	
**HLA sensitisation status pre-transplant**	Non-sensitised	114 (55.9)	66 (51.2)	48 (64.0)	0.19
	Sensitised (cRF 10-84)	54 (26.5)	37 (28.7)	17 (22.7)	
	Highly sensitised (cRF>85%)	36 (17.6)	26 (20.2)	10 (13.3)	
**Type of transplant**	Deceased Donor	172 (84.3)	111 (86.0)	61 (81.3)	0.37
	Living Donor	32 (15.7)	18 (14.0)	14 (18.7)	
**Induction agent**	Alemtuzumab	183 (89.7)	117 (90.7)	66 (88.0)	0.54
	IL2 receptor antagonist	21 (10.3)	12 (9.3)	9 (12.0)	
**Immunosuppression from the time of transplant**	Tacrolimus monotherapy	140 (68.6)	85 (65.9)	55 (73.3)	0.15
	Tacrolimus plus MMF	51 (25.0)	32 (24.8)	19 (25.3)	
	Tacrolimus plus steroids	4 (2.0)	4 (3.1)	–	
	Tacrolimus, MMF plus steroids	9 (4.4)	8 (6.2)	1 (1.3)	
**Diabetes**	No	120 (58.8)	74 (57.4)	46 (61.3)	0.58
	Yes	84 (41.2)	55 (42.6)	29 (38.7)	
**Vaccine type for 1^st^ two doses**	BNT162b2	102 (50.0)	62 (48.1)	40 (53.3)	0.30
	ChAdOx1	101 (49.5)	67 (51.9)	24 (45.3)	
	mRNA-1273	1 (0.5)	–	1 (1.3)	
**Time between last vaccine and transplant**	Days (median)	93 (48-138)	103 (61-153)	64 (43-111)	<0.001
**Infection pre-transplant**	No	114 (55.9)	90 (69.8)	24 (32.0)	<0.0001
	Yes	90 (44.1)	39 (30.2)	51 (68.0)	
Ant-S concentration at transplant*	BAU/ml (median)	799 (97-3154)	232 (45-1694)	2391 (854-5622)	<0.0001
**3^rd^ vaccine**	BNT162b2	182 (89.2)	112 (86.8)	70 (93.3)	0.019
	mRNA-1273	10(4.9)	5 (3.9)	5 (6.7)	
	None	12 (5.9)	12 (9.3)	–	
**4^th^ vaccine**	BNT162b2	100 (49.0)	60 (46.5)	40 (53.3)	0.17
	mRNA-1273	18 (8.8)	15 (11.6)	3 (4.0)	
	None	86 (42.2)	54 (41.9)	32 (42.7)	

*Missing data in 12 patients.

ESKD (End Stage Kidney Disease), cRF (calculated reaction frequency), MMF (mycophenolate mofetil). Bold indicates statistically significant values.

Anti-S results were available on the day of transplant surgery in 120(93.0%) V2-group and 72(96%) V3-group cases. Median [anti-S] were lower pre-transplant in the infection-naïve V2-group, 113(30-555) BAU/ml, compared with the V3-group, 676(261-2903) BAU/ml, p=0.0006. Median [anti-S] remained significantly lower pre-transplant in the infection-experienced V2-group, 1597(358-4614) BAU/ml, compared with the V3-group, 3680(1462-5680) BAU/ml, p=0.024.

### Serological responses to a 3^rd^ vaccine post-transplant in the V2-group

Post-V3 serological assessment was undertaken in 95 V2-group patients who received a third vaccine post-transplantation (PT-V3), 66(69.5%) and 29(30.5%) were infection-naïve and infection-experienced respectively. The median time to PT-V3 was 116(78-150) days post-transplant. Seventeen (25.8%) infection-naïve patients and 22 (75.6%) infection-experienced patients reached the definition of boosted status following PT-V3, p<0.0001. Median [anti-S] PT-V3 was 166(38-720) and 2207(684-5586) BAU/ml in the infection-naïve and infection-experienced individuals respectively, p<0.0001. Infection-naïve patients who had been primed with ChAdOx1 had lower [anti-S] compared with those who have received BNT162b2, following a PT-V3, with median [anti-S] of 77 (38-178) BAU/ml and 315 (194-955) respectively, p=0.015.

**Figure 2:**
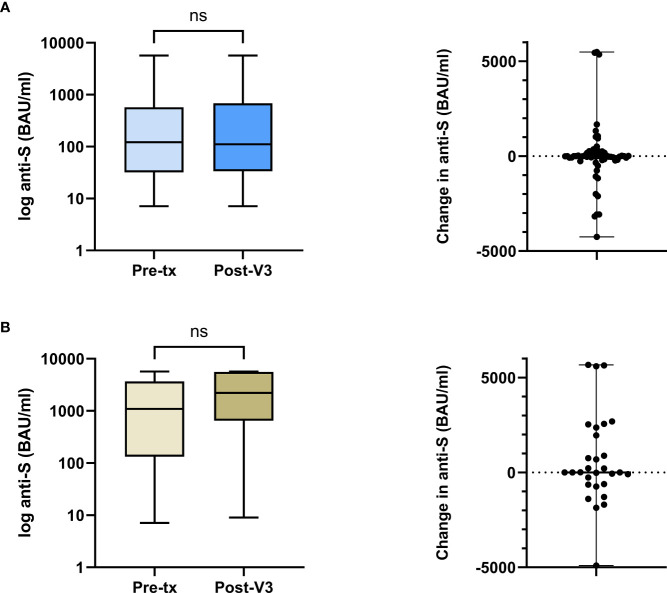
Comparison between pre-transplant and post-V3 anti-S concentrations in the V2-cohort by prior infection exposure. **(A)** In infection naïve patients, the median anti-S pre-transplant and post-V3 did not change, 122 (32-574) and 111 (34-682) BAU/ml respectively, with a median difference of -1 (-182-153) BAU/ml, p=0.78. **(B)** In patients with prior infection, the median anti-S pre-transplant and post-V3 did not change, 1090 (133-3667) and 2207 (650-5618) BAU/ml respectively, with a median difference of 4 (-625-2160) BAU/ml, p=0.26. ns, non-significant.

A paired comparison of pre-transplant and PT-V3 [anti-S] was undertaken in 60 infection-naïve V2-group patients. There was no difference in median [anti-S] pre-transplant and PT-V3, at 122(32-574) and 111(34-682) BAU/ml respectively, with a median difference of -1 (-182-153) BAU/ml, p=0.78, **Figure 2A**. Additional testing was performed in 51/60(85.0%) patients prior to PT-V3 at a median time of 44(32-64) days post-transplant; [anti-S] waned in the absence of infection or vaccination to 40(15-271) BAU/ml, significantly lower than both the corresponding pre-transplant level and following PT-V3 level, p<0.01, **Supplemental Information, Figure S1**.

A further comparison of pre-transplant and PT-V3 [anti-S] was undertaken in 29 infection-experienced patients. There was no difference in median [anti-S] pre-transplant and PT-V3, at 1090(133-3667) and 2207(650-5618) BAU/ml respectively, with a median difference of 4 (-625-2160) BAU/ml, p=0.26, **Figure 2B**.

### Paired comparison of 3^rd^ vaccine responses in transplant patients versus dialysis patients

Anti-S concentrations following a 3^rd^ vaccine dose were analysed in a control group of 63 haemodialysis patients (HD-V3) who contemporaneously received the two vaccines (V1+V2) whilst remaining on the transplant waitlist, of whom 36(57.1%) and 27(42.9%) were infection-naïve and infection-experienced respectively. A comparison of clinical characteristics between this comparator group and the V2-group can be found in the **Supplemental Information, Table S1**.

**Figure 3:**
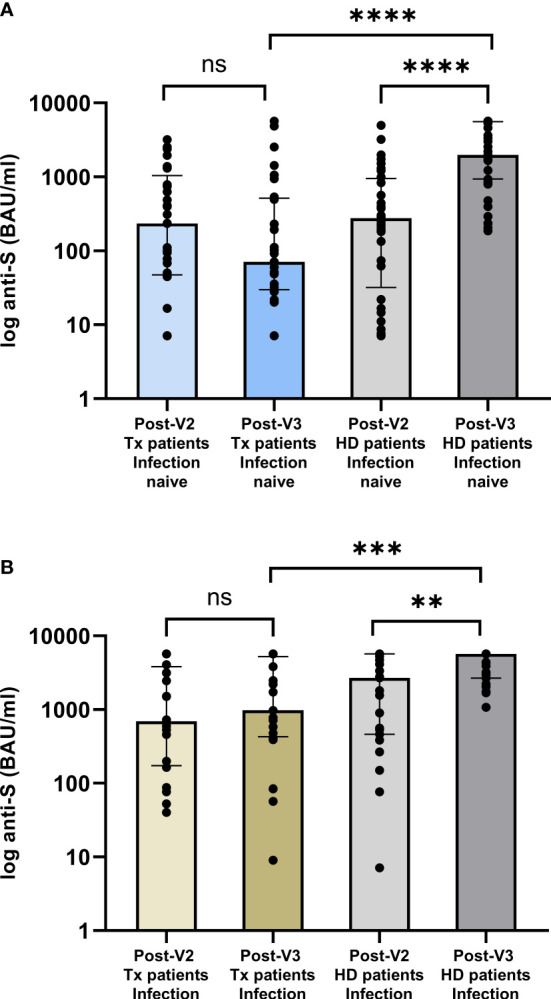
Comparison of serological responses following **(A)** 2^nd^ and 3^rd^ vaccine dose in patients transplanted following **(A)** 2^nd^ vaccine versus those remaining on the waitlist by infection status. In infection naïve individuals, there was no difference between post-V2 anti-concentrations whilst all were on the waitlist, p=0.97. Post-V3, anti-S concentrations were significantly higher in those remaining on the waitlist compared with the V2-group who were subsequently transplanted, median anti-S of 1982 (936-5593) and 71 (30-516) BAU/ml respectively, p<0.0001. **(B)** In patients with prior infection, post-V3 concentrations were significantly higher in dialysis versus transplant patients at 5680 (2681-5680) and 983 (427-5214) BAU/ml respectively, p=0.0006. ****p<0.0001, *** p<0.001, **p<0.01. ns, non-significant.

There was no difference in [anti-S] between the infection-naïve waitlist and pre-transplant V2-group following 2 vaccine doses, with median values of 277(32-952) BAU/ml and 236(48-1046) BAU/ml respectively, p=0.97, **Figure 3A**. However, following a 3^rd^ vaccine dose, [anti-S] was significantly higher in the HD-V3 group, who remained on the waitlist, compared with the PT-V3 group, at 1982(936-5593) and 71(30-516) BAU/ml respectively, p<0.0001. Similarly, whilst there was no difference in [anti-S] in infection-experienced waitlist and pre-transplant V2-group, with median values of 2695(462-5680) and 696(173-3830) BAU/ml respectively, p=0.22; following a 3^rd^ vaccine, [anti-S] was significantly higher in the HD-V3 versus PT-V3 patients at 5680(2681-5680) and 983(427-5214) BAU/ml respectively, p=0.0006, **Figure 3B**.

### Comparison of serological responses following a 4^th^ vaccine dose in V2- and V3-group patients

**Figure 4:**
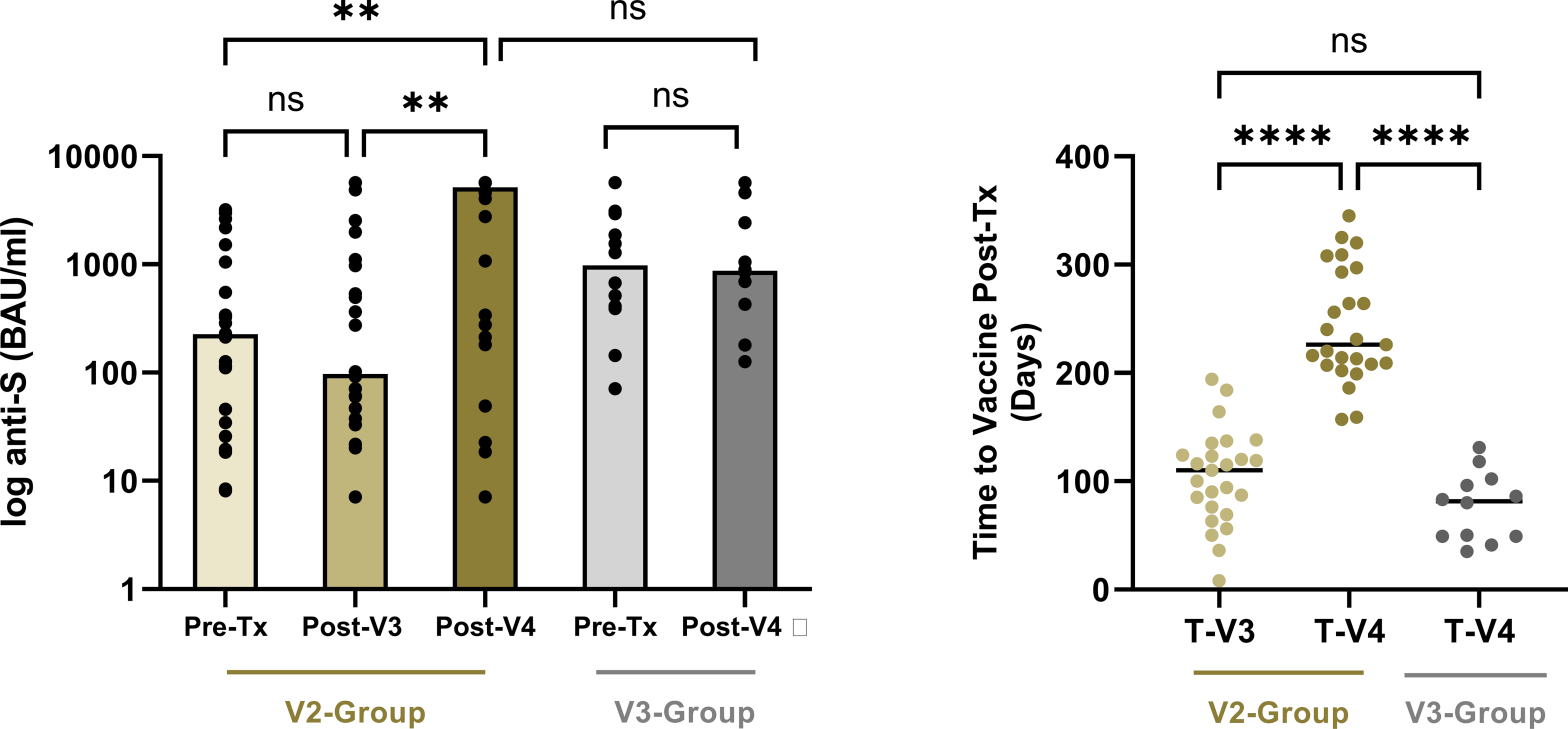
Time to booster doses post-transplant and corresponding paired anti-S concentrations in patients who remained infection naïve. In the V2-group, the median time to the 3^rd^ and 4^th^ vaccine post-transplant was 110 (73-130) and 226 (208-295) days, p<0.001, and the median anti-S concentrations increased from 97 (34-1074) BAU/ml to 5134 (229-5680) post-V3 and V4 respectively, p=0.0016. For the V3-group, there was no increase in anti-S concentrations from the time of transplant to post-V4, with median values of 981 (396-2666) BAU/ml and 871 (242-2092) and respectively, p=0.62. **p<0.01; ****p<0.0001. ns, non-significant.

From 75 V2-group patients who received 2 vaccines (V3 and V4) post-transplant, paired serological testing was available in 36/75 (48.0%) patients at the time of transplantation, following -V3 and following -V4. The median time to PT-V4 in these 36 patients was 226(208-295) days. The corresponding median time to serological testing following PT-V3 and PT-V4 was 33(25-42) and 30(22-40) days respectively, p=0.56. At the time of testing PT-V4, 24/36(66.7%) patients remained infection-naïve, 16(66.7%) of whom met the serological criteria for a boosted status, compared with only 7(29.2%) following PT-V3, p=0.01. Median [anti-S] in infection-naïve patients following PT-V4 versus PT-V3 was 5134 (229-5680) versus 97(34-1074) BAU/ml respectively, p=0.0016, **Figure 4**. Only 1/12 (8.3%) infection-experienced patients did not meet boosted status PT-V4.

From 43 V3-group patients who received a 4^th^ vaccine post-transplant (having received V1-3 pre-transplant), paired serological testing at the time of transplant and post-V4 was available in 35(81.3%). The median time to PT-V4 in the V3-group was 82(49-101) days, which was significantly shorter than the comparator V2-group who received PT-V4 at median time of 226(208-295) days, p<0.0001 post-transplant, **Figure 4**. Only 12/35(34.3%) V3-group patients were considered infection-naïve at the time of sampling PT-V4, at a median of 34 (21-42) days post vaccination. Seven of 12 (58.3%) patients were considered to have boosted status, although there was no significant difference in [anti-S] PT-V4 compared with at the time of transplant for these patients, with median values of 871(242-2092) and 981(396-2666) BAU/ml respectively, p=0.62, **Figure 4**. Twenty-three (65.7%) V3-group patients were infection-experienced at the time of testing PT-V4, of whom 17(68.0%) were considered boosted. There was no difference in [anti-S] PT-V4 compared with pre-transplantation, at 5477(690-5680) and 239(1089-5622) BAU/ml respectively, p=0.31.

### SARS-CoV-2 infections and outcomes post-transplant

Ninety of all 204 (44.1%) patients were infection-experienced at the time of transplant. Seventy-seven patients (37.8%) were diagnosed with infection post-transplant, of which 24/77 (31.2%) cases were re-infections. Despite high reinfection rates, patients with infection exposure pre-transplantation were less likely to become infected post-transplant compared with infection-naïve patients, with 24/90(26.7%) and 53/114(46.5%) infections respectively; OR: 0.42 (0.23-0.76), p=0.004. The majority of the V2-group, 87/129 (67.4%), were transplanted whilst the Delta variant was dominant. In the V3-group, 47/75 (62.7%) patients were transplanted during the Omicron period. There was no difference in infection-free survival post-transplant between the 2 groups at the end of follow up, p=0.09, **Supplemental Information, Figure S2**. However, 51/55 (92.7%) of infected V2-group patients were diagnosed during the Omicron period, as were all V3-group patients.

Patients who were diagnosed with infection post-transplant had lower [anti-S] at the time of transplant compared with those who remained infection-free, with concentrations of 515(38-1847) and 1281(178-3925) BAU/ml respectively, p=0.004. Of patients who were diagnosed with infection post-transplant, 51/77 (66.2%) had received an additional vaccine dose during the post-transplant period. Five of 77 (6.5%) patients who were diagnosed with infection post-transplant died, of whom 3 (3.9%) were within 28 days of COVID infection. All five cases had PCR confirmed infection, all occurring during the Omicron period, 4 with available results confirming Omicron by genotyping. Twenty-three of 77 (29.9%) patients with post-transplant infection were hospitalised at the time or within 28 days following diagnosis; 5/23(21.7%) were infected following nosocomial transmission. Primary indication for admission may be found in the **Supplementary Information, Table S2**. By comparison, of the waitlist controls, 18/63 (28.6%) were diagnosed with infection post at least 3 doses of vaccine, of which there were no admissions and no deaths recorded.

Fifty patients had paired serology pre- and post-infection. Thirty-three (66.0%) had no interval vaccination, of whom 13 (39.4%) received monoclonal antibody treatment (mAb). Seventeen (34.0%) had an interval vaccine dose in addition to infection, of whom 5/17 (29.4%) received monoclonal antibody treatment, two casirivimab plus imdevimab and 15 sotrovimab. There was no significant difference in time to anti-S testing post-infection in the vaccine+mAb+, vaccine+mAb-, vaccine-mAb+ and vaccine-mAb- patients at a median of 35 (14-143), 72 (45-99), 65 (28-89) and 61 (36-143) days post-infection respectively, p=0.77. There was also no difference in [anti-S] between the groups, with median levels of 2370(1418-5680), 3703(129-5680), 3386 (2487-5680) and 1937(673-4903) BAU/ml respectively, p=0.27, **Supplemental Information, Figure S3**. All groups had a significant increase in anti-S compared with pre-transplant except for the vaccine+mAb+ group, in whom [anti-S] rose from 39 (7.1-879) to 2370 (1418-5680), p=0.13, **Supplemental Information, Figure S4**.

## Discussion

This study has shown both important confirmatory and novel findings. Firstly, prevalence of breakthrough SARS-CoV-2 infection in *de novo* transplant recipients is high, with nosocomial transmission common. Pre-transplant infection exposure and [anti-S] at the time of transplant predicted post-transplant infection risk. There was no increase in [anti-S] in transplant patients receiving either V3 or V4 in the first few months post-transplant. However, there was a significant increase in [anti-S] in patients who received a booster after a median time of 6 months, suggesting timing was important. Finally, mAb therapy did not appear to negatively impact on longitudinal [anti-S] in those patients who were diagnosed with infection and vaccinated. However, enhanced [anti-S] were seen in those treated for infection in the absence of a booster vaccine

Despite infection being a major cause of morbidity and mortality, there is sparse high quality evidence on the optimal dosing and timing of vaccination post-transplantation (10). Recent influenza vaccination studies have led to the use of increased dosing and the administration of vaccines as little as 4 weeks post-transplant (9, 12, 13). However, mechanistic work, and the correlation between immunogenicity and effectiveness is lacking. Although the pandemic has brought a considerable amount of data on vaccine immunogenicity and effectiveness of SARS-CoV-2 vaccines to the fore, little has been reported on those patients primed pre-transplant but requiring boosters in the early post-transplant period. Currently, booster schedules are an evolving field in the general population, with reactive strategies being implemented in response to real-time effectiveness data coupled with *in-vivo* and *in-vitro* immunogenicity data to circulating variants (4, 5). This unstable dynamic makes guidance for the immunosuppressed even more challenging. In the UK, immunosuppressed patients will be offered their 6^th^ SARS-CoV-2 vaccine from September 2022, although there will be considerable heterogeneity in the immune repertoire in immunosuppressed patients depending on clinical characteristics, type of immunosuppression, prior infection, number of vaccines, vaccine type and timing related to immunosuppression. Although in general, vaccination received prior to immunosuppression will evoke more immunogenic responses, it should be considered that for patients with end stage kidney disease (ESKD) who have been transplanted during the pandemic, it is likely that all vaccinations were received whilst they were at advanced chronic kidney disease stages. It is recognised that people with ESKD requiring dialysis have weaker immune responses to SARS-CoV-2 vaccines. Consequently, it is likely, even prior to iatrogenic immunosuppression, that pre-transplant patients are less protected than the general population (8, 14, 15).

Although the seroresponse rate and [anti-S] reported in this study appear to be superior to immunogenicity data reported in transplant recipients who were primed with SARS-CoV-2 vaccines post-transplant, it may be argued that new transplant patients still require bespoke protection strategies (7). We know that in the pre-vaccination era, mortality risk was greatest in patients in the early post-transplant period, which is likely to be related to an enhanced immune suppressed state (16–18). Therefore, it may be hypothesised that infections may have a greater impact in vaccinated individuals in the early period post-transplant too, although to our knowledge, there are no reported data on this comparison. New transplant patients are obligated to attend healthcare environments very frequently in the first year after surgery. As communities return to ‘normal living’ and infection rates remain high, these frequent hospital visits may undermine attempts to mitigate the risk of contracting infection with physical protective measures. Indeed, we report cases of nosocomial transmission during the primary transplant episode. Whilst hospitalisation and mortality are considered important outcome metrics for policy makers, for transplant patients other factors such as rejection and impact on long term allograft function will be equally important. This data is not fully appreciated yet.

Whilst vaccination scheduling and timing of transplantation could be planned for living donor recipients, for deceased donor recipients this is not always possible. Additionally, booster programmes are not produced by transplant centres, but rather national policy makers. Therefore, if only a given number of vaccines are permissible by policy in the first 6 months post-transplant, timing of that booster dose will be important. However, other than vaccination, many countries are advocating the use of passive pre-exposure prophylaxis with monoclonal antibodies for people with weakened immune systems (19, 20). Although the monoclonal antibody of choice will need to change in response to evolving variants, administration of antibody at the time of discharge from hospital after transplantation surgery seems a pragmatic compromise, with provision of booster doses of vaccine deferred to a minimum of 3 to 6 months. Of course this advice may change if data emerges on adverse outcomes such as negative impact of monoclonal therapy on immune response to subsequent vaccination (21).

This study has limitations which will restrict robust conclusions. Perhaps most important of which is the non-uniform timing of sampling performed, which was ameliorated using paired samples. The sample sizes were too small to adjust for baseline clinical characteristics, which again was partially compensated for using within-subject comparisons. The study would have been strengthened by incorporating cellular responses and assessing neutralising capability of antibodies. In addition, the study included patients transplanted over a one-year period, consisting mainly of the period dominated by the Delta and Omicron variants, with different prevalent rates of community infection throughout. Therefore, effectiveness data has been limited to descriptive only, with a greater focus on the serological responses to vaccines, infection and treatment. However, despite these limitations, to our knowledge this is the first study to describe serological responses in relation to booster vaccines given post-transplant, and provides preliminary evidence on optimal timing of boosters, or at least the potential need for additional doses or passive immunity in this population during the first year.

In conclusion, this study has shown that transplant recipients who are primed pre-transplant mount attenuated serological responses to booster doses of SARS-CoV-2 vaccines in the early transplant period. Responses improve with subsequent doses given at longer periods from the time of transplant, with serological responses seen by 6 months post-transplant. Anti-S concentrations at the time of transplant predict infection in our cohort, and we would re-iterate the importance of vaccination pre-transplant. However, it may be prudent to provide prophylactic monoclonal antibody to cover the first 3 to 6 months post-transplant whilst the pandemic continues, as this is when there is high intensity exposure to health care settings, patients are highly immunosuppressed and boosters the least immunogenic. Whatever the policy applied in different countries, we hope the pandemic precipitates much needed prospective studies of optimal dosing and timing of vaccines in solid organ transplant recipients, to maximise protection in these patients.

## Data availability statement

The datasets presented in this article are not readily available because data was obtained from routinely collected clinical information. Requests to access the datasets should be directed to m.willicombe08@imperial.ac.uk.

## Ethics statement

The studies involving human participants were reviewed and approved by Health Research Authority, Research Ethics Committee (Reference: 20/WA/0123). Written informed consent for participation was not required for this study in accordance with the national legislation and the institutional requirements.

## Author contributions

SG, PM, TT and MW contributed to the concept of the study. All authors contributed to the acquirement of data. SG, PM and MW wrote the first draft, and All authors contributed to the article and approved the submitted version.
