# Supplementary material for: Lack of seroresponse to SARS-CoV-2 booster vaccines given early post-transplant in patients primed pre-transplantation
Source: Front Immunol. 2023 Jan 16;13:1083167. doi: 10.3389/fimmu.2022.1083167 (PMC9885043; doi:10.3389/fimmu.2022.1083167)

**Supplemental Information**

**Table S1. Characteristics of wait list controls and V2-group**

| Characteristics | | V2-Group  N=129 (%) | Wait-list Group  N=63(%) | P value |
| --- | --- | --- | --- | --- |
| Gender | Male  Female | 74 (57.4)  55 (42.6) | 44 (69.8)  19 (30.2) | 0.09 |
| Age at transplantation | Years (Median) | 55 (45-65) | 55 (47-65) | 0.52 |
| Ethnicity | White  Black  Indoasian  Other | 38 (29.5)  18 (14.0)  53 (41.1)  20 (15.5) | 25 (39.7)  10 (15.9)  20 (31.7)  8 (12.7) | 0.47 |
| Cause of ESKD | Polycystic kidney disease  Glomerulonephritis  Diabetic nephropathy  Urological  Unknown  Other | 13 (10.1)  26 (20.2)  45 (34.9)  8 (6.2)  27 (20.9)  10 (7.8) | 2 (3.2)  17 (27.0)  20 (31.7)  3 (4.8)  14 (22.2)  7 (11.1) | 0.51 |
| Immunosuppressed | No  Yes | -  129 (100) | 9 (14.3)  54 (85.7) | <0.0001 |
| Diabetes | No  Yes | 74 (57.4)  55 (42.6) | 32 (50.8)  31 (49.2) | 0.39 |
| Vaccine type for 1^st^ 2 doses | ChAdOx1  BNT162b2 | 67 (51.9)  62 (48.1) | 40 (63.5)  23 (36.5) | 0.13 |

**Figure S1. Paired anti-S concentrations pre-transplant, post-transplant but pre-V3 and post-V3, in 51 infection naïve individuals**

Median anti-S concentrations were significantly lower at a median of 44 (32-64) days post-transplant compared with pre-transplant at 40 (15-271) versus 111 (31-583) BAU/ml respectively, p<0.0001. Post-transplant but pre-V3 anti-S was also lower than post-V3, 112(38-945) BAU/ml, p=0.004.


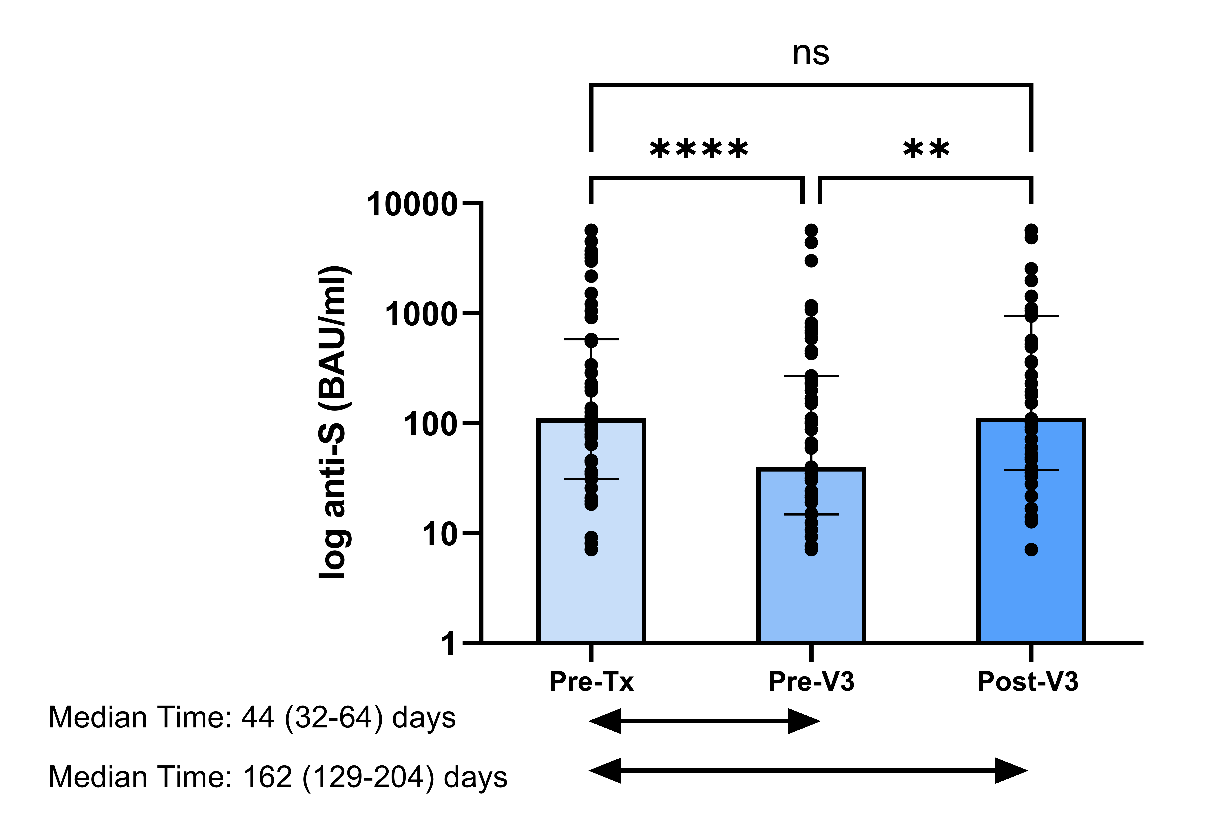


**Figure S2. Infection free survival in V2- versus V3-group patients**

There was no difference in infection free survival post-transplant between V2- and V3-group patients, n=0.09 (log-rank). Of 129 V2-group patients, 38 (29.5%), 87 (67.4%) and 3/129 (2.3%) were transplanted when the alfa, delta and omicron variants were dominant respectively; whilst 28/75 (37.3%) and 47 (62.7%) of the V3-group patients were transplanted during the delta and omicron periods respectively.


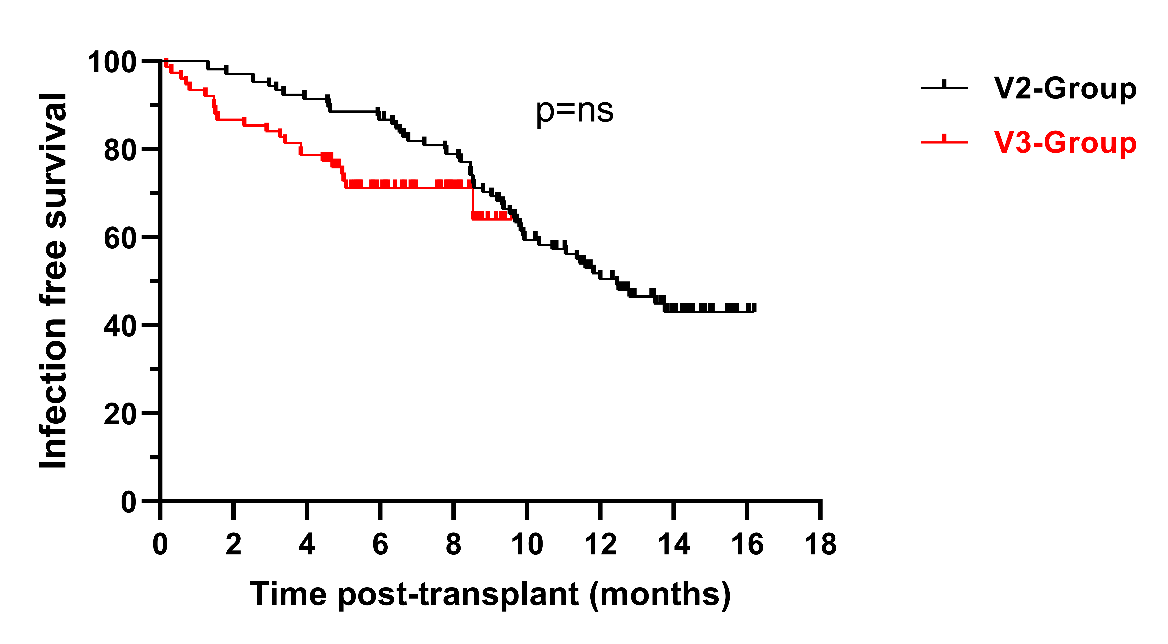


**Table S2. Indications for admission to hospital following a diagnosis of COVID**

| **GROUP** | **COVID PRE-TRANSPLANT** | **NUMBER OF VACCINES AT TIME INFECTION** | **TIME TO ADMISSION POST-DIAGNOSIS** | **REASON FOR ADMISSION** | **OTHER INFECTION** | **SARS-CoV-2 INFECTION EPISODE** |
| --- | --- | --- | --- | --- | --- | --- |
| V2 | NO | 3 | NOSOCOMIAL | CARDIAC EVENT |  | 1 |
| V2 | NO | 3 | NOSOCOMIAL | GRAFT DYSFUNCTION |  | 1 |
| V3 | YES | 3 | NOSOCOMIAL | TRANSPLANT EPISODE |  | 2 |
| V3 | YES | 3 | NOSOCOMIAL | TRANSPLANT EPISODE |  | 2 |
| V3 | YES | 3 | NOSOCOMIAL | TRANSPLANT EPISODE |  | 3 |
| V2 | NO | 3 | 14-21 DAYS | INFECTION | PCP | 1 |
| V2 | YES | 3 | 14-21 DAYS | HAEMOLYTIC ANAMEIA |  | 2 |
| V2 | YES | 2 | 14-21 DAYS | INFECTION | URINE | 2 |
| V2 | NO | 4 | ≤7 DAYS | INFECTION | INFECTION | 1 |
| V3 | YES | 3 | ≤7 DAYS | INFECTION | WOUND | 2 |
| V3 | YES | 3 | ≤7 DAYS | TRANSPLANT EPISODE |  | 2 |
| V2 | NO | 3 | ≤7 DAYS | INFECTION | URINE | 1 |
| V3 | NO | 4 | ≤7 DAYS | INFECTION | COVID | 1 |
| V2 | NO | 2 | ≤7 DAYS | GRAFT DYSFUNCTION |  | 1 |
| V3 | NO | 3 | ≤7 DAYS | INFECTION | COVID | 1 |
| V3 | YES | 3 | ≤7 DAYS | INFECTION | BACTERAEMIA | 2 |
| V2 | NO | 2 | ≤7 DAYS | INFECTION | URINE | 1 |
| V3 | YES | 3 | ≤7 DAYS | GRAFT DYSFUNCTION |  | 2 |
| V2 | NO | 3 | ≤7 DAYS | GRAFT DYSFUNCTION |  | 1 |
| V3 | NO | 3 | ≤7 DAYS | INFECTION | BACTERAEMIA | 1 |
| V2 | NO | 2 | ≤7 DAYS | INFECTION | COVID | 1 |
| V3 | YES | 3 | ≤7 DAYS | PULMONARY EMBOLUS |  | 2 |
| V2 | NO | 3 | ≤7 DAYS | INFECTION | WOUND | 1 |

**Figure S3. Post-infection anti-S concentrations by interval vaccine status and monoclonal antibody treatment**

Anti-S concentrations post-infection in the vaccine+mAb+, vaccine+mAb-, vaccine-mAb+ and vaccine-mAb- patients were tested at a median time of 35 (14-143), 72 (45-99), 65 (28-89) and 61 (36-143) days respectively, p=0.77. There was also no difference in anti-S concentrations between the groups, with median levels of 2370 (1418-5680), 3703 (129-5680), 3386 (2487-5680) and 1937 (673-4903) BAU/ml respectively, p=0.27.


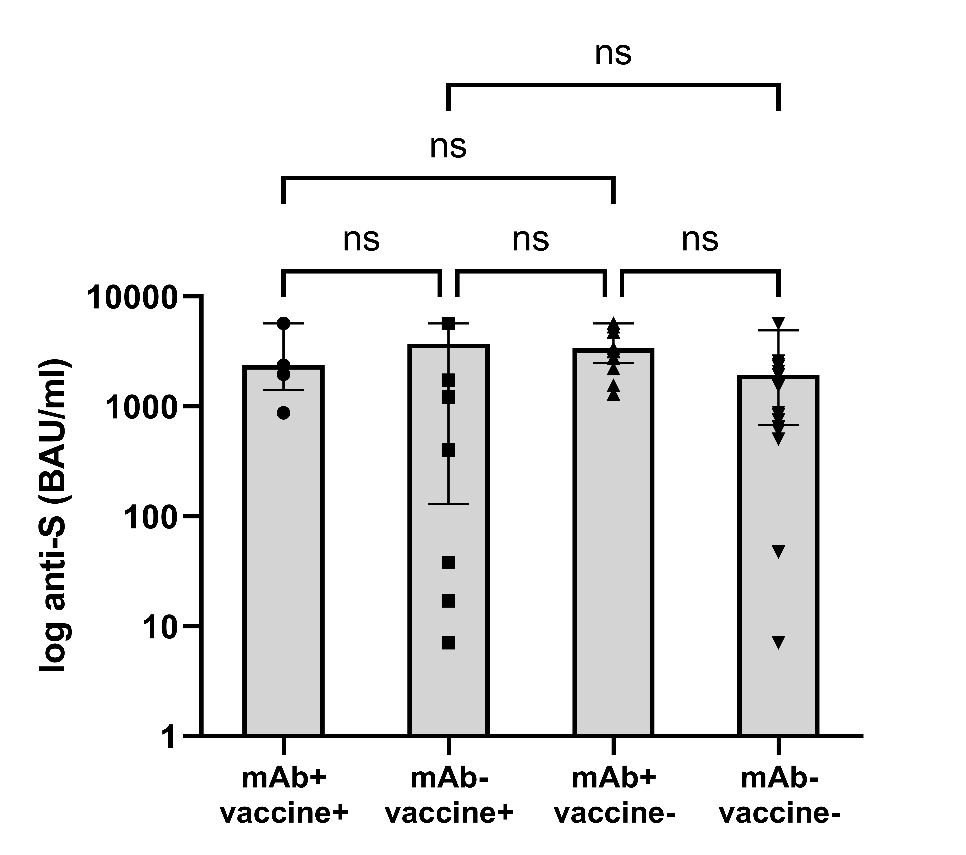


**Figure S4. Comparison of pre-transplant and post-infection anti-S concentrations by interval vaccine status and monoclonal antibody therapy**

Paired pre-transplant and post-infection concentrations in patients receiving a. no interval vaccine or mAb treatment rose from 838 (101-1867) to 1940 (751-5680) BAU/ml, p=0.007, b. no interval vaccine but did receive mAb treatment, 213 (111-1746) to 3283 (2626-5680), p=0.0098, c. an interval vaccine plus mAb treatment, 39 (7.1-879) to 2370 (1418-5680) BAU/ml, p=0.13 and d. an interval vaccine but no mAb treatment, 127 (16-1623) to 3703 (129-5680) BAU/ml, p=0.0034.


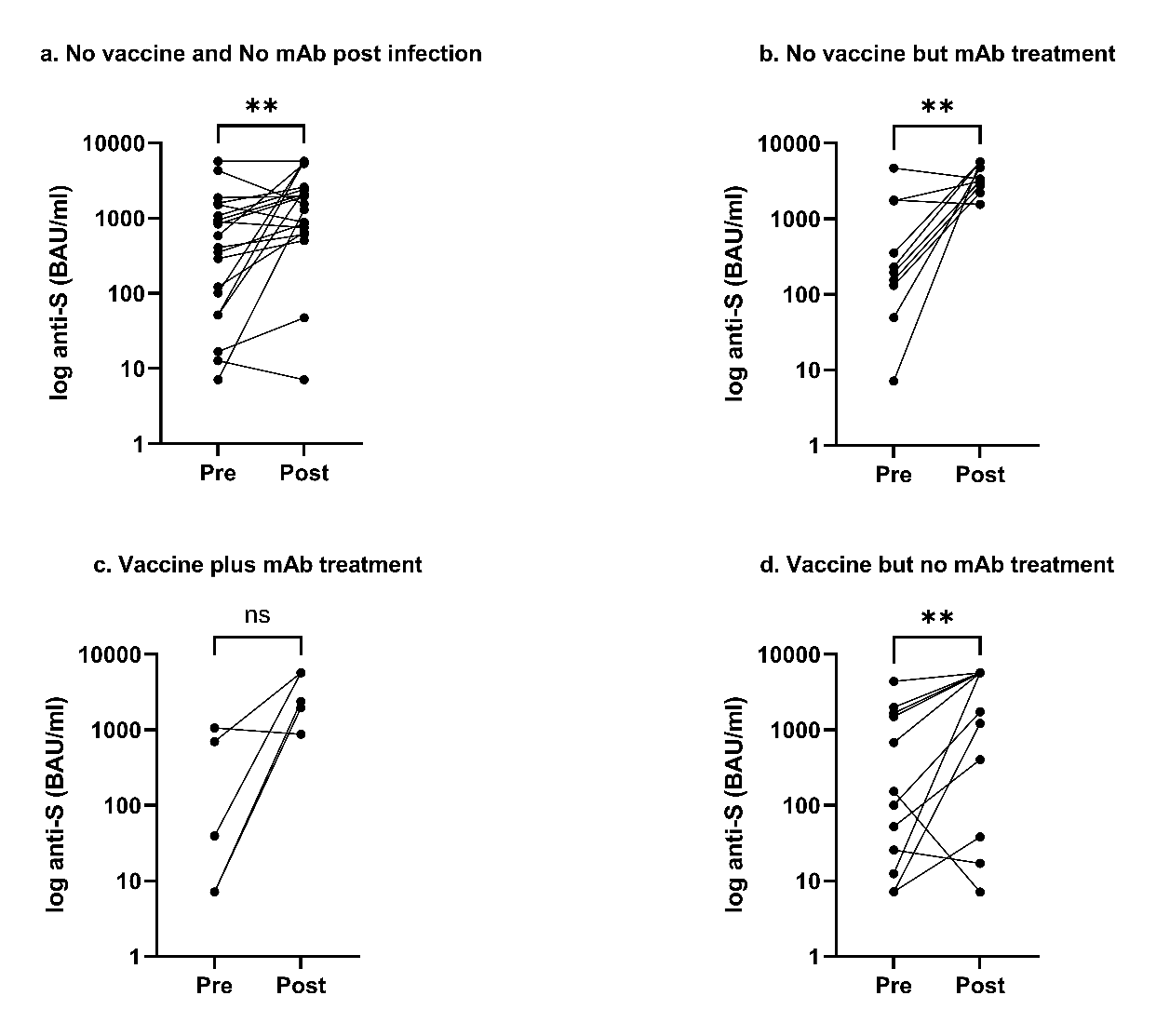

Supplement: Supplementary file 1 [file DataSheet_1.docx]
